# Utility of the first few100 approach during the 2009 influenza A(H1N1) pandemic in the Netherlands

**DOI:** 10.1186/2047-2994-1-30

**Published:** 2012-09-21

**Authors:** Arianne B van Gageldonk-Lafeber, Marianne AB van der Sande, Adam Meijer, Ingrid HM Friesema, Gé A Donker, Johan Reimerink, Mirna Robert-Du Ry van Beest Holle, Jan M Prins, Leslie Isken, François G Schellevis, Mariken IM van der Lubben

**Affiliations:** 1 National Institute for Public Health and the Environment (RIVM), PO box 1 3720 BA, Bilthoven, the Netherlands; 2 Utrecht University Medical Center, Julius Centre, Utrecht, the Netherlands; 3 Netherlands institute for health services research (NIVEL), Utrecht, the Netherlands; 4 Department of Internal Medicine, Division of Infectious Diseases, Tropical Medicine and AIDS, Academic Medical Center, Amsterdam, the Netherlands; 5 Department of General Practice/EMGO + Institute, VU University Medical Center, Amsterdam, the Netherlands

## Abstract

**Background:**

To guide policy and control measures, decent scientific data are needed for a comprehensive assessment of epidemiological, clinical and virological characteristics of the First Few hundred (FF100) cases. We discuss the feasibility of the FF100 approach during the 2009 pandemic and the added value compared with alternative data sources available.

**Methods:**

The pandemic preparedness plan enabled us to perform a case–control study, assessing patient characteristics and risk factors for experiencing symptomatic influenza A(H1N1)2009 infection and providing insight into transmission. We assessed to what extent timely and novel data were generated compared to other available data sources.

**Results:**

In May-December 2009, a total of 68 cases and 48 controls were included in the study. Underlying non-respiratory diseases were significantly more common among cases compared to controls, while a protective effect was found for frequent hand washing. Seroconversion was found for 7/30 controls (23%), and persisting high titers for 4/30 controls (13%). The labour-intensive study design resulted in slow and restricted recruitment.

**Conclusions:**

The findings of our case–control study gave new insights in transmission risks and possible interventions for improved control. Nevertheless, the FF100 approach lacked timeliness and power due to limited recruitment. For future pandemics we suggest pooling data from several countries, to enable collecting sufficient data in a relatively short period.

## Background

The worldwide increase in the incidence of influenza caused by avian influenza viruses since 1997, both in poultry and humans, introduced the potential for another influenza pandemic and the need for pandemic preparedness plans [1-4]. Comprehensive assessment of the First Few Hundred (FF100) cases to timely characterise clinical, virological and epidemiological features and for risk factor information is an important part of these plans. Despite the continuing threat of avian influenza viruses, the first official influenza pandemic in the 21st century was caused by the so-called influenza A(H1N1) 2009 virus. In the Netherlands, as elsewhere, initial rapid assessments of the impact of this new influenza virus on the human population have been based on case-studies of the first notified laboratory confirmed cases [5,6]. These studies provided vital information to guide management, but more standardised and detailed information was still needed to guide control activities, to underpin policy decisions and for communication with the general public and the media. The Dutch pandemic preparedness plan included a generic study protocol and questionnaires to rapidly collect detailed key epidemiological, clinical, virological and immunological data of a limited number (approximately one hundred) of the earliest patients and their close contacts. The design of this FF100 cases and contacts approach enabled a case–control study aiming at identifying patient characteristics and risk factors for experiencing a symptomatic influenza A(H1N1) 2009 infection in the general Dutch population, and made it possible to get the insight into the transmission of the influenza virus. This is in contrast to other studies and FF100 approaches, which focused on cases only and were therefore dependant on ecological analysis for risk factor analysis. 

**Table 1:** **Study schedule of the case–control study in the period before and after the change of notification criteria for influenza A(H1N1) 2009 virus infection at the 15**^**th**^**of August 2009, the Netherlands**

		***Day 0***	***Day 5 range [4-6]***	***Day 10 range [9-11]***	***Day 30 range [28–35]***
*Up to 15 August 2009*		**visit1**	**visit2**	**visit3**	**visit4**
case patients	nose- and throat swab^*^		X	X	
	venipuncture	X^^^		X	X
control subjects	nose- and throat swab	X			
	venipuncture	X		X	X
*From 15 August 2009*		**GP visit**	**visit1**	**visit2**	**visit3**
case patients	nose- and throat swab	X^^^	X	X	
	venipuncture	X^^ #^	X	X	X
control subjects	nose- and throat swab		X		
	venipuncture		X	X	X

^*^ The diagnostic nose- and throat swab on which the patients were included in the study was taken by the physician of the municipal health service and is not entered in the schedule.

^ For hospitalised cases the first visits, until the moment of discharge, were scheduled in hospital.

^#^ The GP took a finger prick instead of a venipuncture.

This report summarises the findings from the case–control study with respect to risk factors for symptomatic infection and transmission. Moreover, the FF100 cases and contacts approach is discussed in terms of the feasibility during the 2009 pandemic and the added scientific value compared with alternative data sources available. We will go into the difficulties of performing this approach real time and the lessons learned.

## Methods

### Study design

A comprehensive generic study protocol for detailed data collection was written following the 2003 outbreak of influenza A(H7N7) among poultry in the Netherlands [7,8], and approved beforehand by the Medical Ethical Review Committee of the University Medical Center Utrecht in 2007. In May 2009, the protocol was adapted to the situation of the pandemic threat at that time and again approved by the same Medical Ethical Review Committee. The study started in June 2009, and was performed by the Centre for Infectious Disease Control (CIb) of the National Institute for Public Health and the Environment (RIVM), in collaboration with Public Health Services (PHS), a network of academic medical centres and the network of general practitioners (GPs) from the Continuous Morbidity Registration Sentinel General Practice Network of the Netherlands institute for health services research (NIVEL).

### Cases and controls

Initially, laboratory-confirmed cases as well as controls were recruited via the PHS using the national mandatory notification system. Since the 15^th^ of August, the notification criteria were limited from all possible, probable and laboratory-confirmed cases to hospitalised or deceased laboratory-confirmed cases. Therefore, the recruitment of study participants was continued in collaboration with GPs from the Continuous Morbidity Registration Sentinel General Practice Network of the NIVEL [6]. The selection and inclusion of cases is described in more detail by Friesema et al [9]. Contacts were defined as persons who had close contact with the case in the early stage of infection, and who had no symptoms of ILI at the time of inclusion in the study. They were approached for participation in the study at the moment patients were informed about the study. These contacts were considered as controls matched for exposure to influenza A(H1N1) 2009 virus. Controls developing symptoms of ILI during the period of follow up were maintained as controls according to the study design.

### Data collection

After consenting, both cases and controls were visited by a research nurse at day 0 (within 8 days from the onset of disease). During this first visit the study objectives were further clarified, the case and control information was handed over, and written informed consent was obtained from both the case and control(s). As shown in Table 1, data collection was carried out at days 0, 5, 10 and 30. From the 15^th^ of August onwards, the first GP consultation was counted as the day 0-visit for cases and the first home visit for controls was scheduled at day 5. For cases as well as controls, the number and type of samples taken before and after the 15^th^ of August were the same, with the exception of an extra blood sample (finger prick) for cases taken at the GP visit. The same schedule was also applied for hospitalised cases and their controls. During hospitalisation, the study was carried out in the hospital by the attending physician. After discharge, the remaining home visits were taken over by a research nurse.

Both cases and controls were asked to complete a detailed questionnaire. This questionnaire included questions about demographics, medical history, use of medication, exposure to influenza A(H1N1) 2009 virus, symptoms of the current episode, and hygiene aspects.

Real-time RT-PCR for detection of pandemic influenza A(H1N1) 2009 virus in combined nose and throat swabs and serology was done as described previously [10,11]. Seroconversion was defined as a change in titre from no hemagglutination inhibition to a titre ≥1:40, or as having a four-fold or greater rise in titres between two successive samples.

### Statistical analyses

Multivariate logistic regression analysis was used to examine whether patient characteristics and/or potential risk factors were associated with a laboratory-confirmed symptomatic infection. The dependent variable was symptomatic disease (i.e. being a case or control). Variables with a P-value ≤ 0.10 in the univariate model were included in the multivariate model. Backward selection was used to identify covariates that were independently associated with symptomatic disease. Significant odds ratios (ORs) were presented with 95% confidence intervals (95% CI).

Insight in transmission was obtained by studying the serology results for controls. These analyses were restricted to controls of whom at least two blood samples were available. Persisting high titers (≥ 1/40) in the first and following blood samples were considered as a proxy for exposure to the same source as the case, while seroconversion was considered as a proxy for secondary transmission whereby the case acts as probable source for the control.

With respect to the feasibility of our study design during the 2009 pandemic, we assessed the number and timing of inclusion of patients willing to participate in the study versus the total number of notified influenza A(H1N1) 2009 virus infections in relation to the timing of notifications. Furthermore the age distribution of included patients was compared with that of the notified patients. The added value of the used approach was explored by comparing the findings of the case–control study with alternative data sources. Statistical analyses were performed using SAS version 9.2 (SAS Institute).

## Results

**Figure 1:**
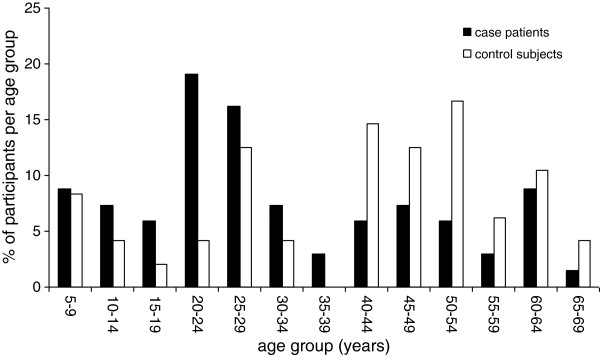
Age distribution of cases and controls of the case–control study during the influenza A(H1N1) 2009 pandemic in the Netherlands, 2009.

Between June and December 2009 a total of 76 of 120 invited patients were willing to participate in the study (63%). Written informed consent and a completed questionnaire were received for 68 of these cases, as well as for 48 controls. The response rate for controls could not be assessed, since no information about the number of approached controls was available. Next to the questionnaire, at least one combined nose and throat swab was available for all cases and at least one blood sample for 32 cases (47%). For the controls these numbers were 34 (71%) and 33 (69%), respectively. The age distribution of cases and controls is shown in Figure 1.

The median age of cases (26 years, range: 5–67 years) was significantly lower than that of controls (45 years, range: 7–67 years). Underlying lung disorders, including asthma, COPD, and cystic fibrosis, were reported by 28% of the cases and 25% of the controls (p = 0.7). Underlying non-respiratory diseases, including various disorders like cardiovascular disorders, immunological disorders and diabetes mellitus, were reported by 18% of the cases and 5% of the controls (p = 0.04).

### Risk factors

Cases and controls were compared with respect to reported medical history, smoking, antiviral treatment, seasonal influenza vaccination status, and current hygiene aspects to assess which variables were independently associated with symptomatic disease. Multivariate logistic regression analyses, adjusted for age and gender, showed that underlying non-respiratory diseases (including cardiovascular disorders, immunological disorders and diabetes mellitus) were significantly more common among cases compared to controls (OR = 9.7; 95%CI: 1.6-57.9). Next to this, a protective effect of frequent (≥8 times a day) hand washing was found (OR = 0.37; 95%CI: 0.15-0.90).

### Transmission

Seroconversion was found for seven of the 30 controls (23%), of whom at least two blood samples (minimal 8 days apart) were available. For three of these seven controls the PCR of the combined nose and throat swab, taken at the inclusion in the study, was positive.

Persisting high titers in the first and following blood samples were found for four of the 30 controls (13%). For one of them the PCR, taken at the inclusion in the study, was positive. For a total of 19 controls (63%) no serological response was found.

### FF100 cases and contacts approach

**Figure 2:**
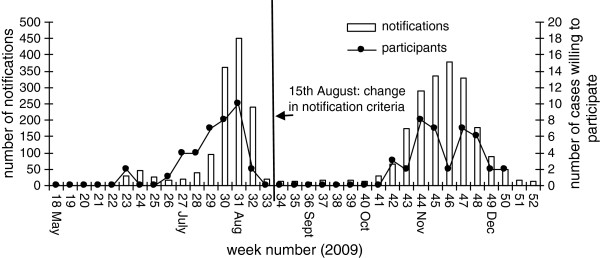
Number of notified patients and number of cases willing to participate in the case–control study during the influenza A(H1N1) 2009 pandemic in the Netherlands, 2009.

Inclusion of the required number of cases and contacts during the period of influenza activity in the Netherlands lagged behind the notified cases reported to the national mandatory notification system. By the time the first hundred patients were notified, 2 cases were recruited, and by the time 200 patients were notified, 14 cases were recruited. Figure 2 shows the number of included patients willing to participate in relation to the number of notified patients.

About 50% of the cases were included via the PHS before the 15^th^ of August. The remaining 50% was included via the GPs from the NIVEL network. Three of the participating cases were admitted to hospital, of which two were included through the participating academic medical centres. The inclusion of hospitalised cases could only start a median of 153 days after the approval of the baseline protocol, because it was necessary to obtain approval of the study protocol by the Medical Ethical Review Committees of the individual academic centres.

All age groups were represented in the case–control study (Figure 1), and the age distribution of the included cases corresponds to that of the first 115 laboratory-confirmed cases in the Netherlands [5].

The control/case ratio in the case–control study was 0.7 (48/68), indicating we included less than one control per case. The inclusion of children as control was in particular difficult, as indicated by the significantly higher median age of controls compared to cases.

## Discussion

The Dutch FF100 case–control was unique in including controls alongside cases and observed an increased risk of symptomatic infection among those with non-respiratory underlying conditions, while frequent hand washing was found to be protective. In addition, serological results indicated that nearly a quarter of exposed contacts had evidence of secondary transmission rate. However, the labour-intensive design was logistically extremely challenging during times of intense work pressure, resulting in slow and limited recruitment, and results might have been biased by the low number of included cases and controls. Despite intensive preparedness planning the added value of the comprehensive assessment of the FF100 cases and contacts during the 2009 influenza pandemic in the Netherlands was less than expected due to limited number of participants underlying the novel observations and the delay in recruitment of both cases and controls.

The results of our study suggest an increased risk for symptomatic infection in patients with underlying non-respiratory diseases. This is in contrast with several other studies observing that established risk factors for complications of seasonal influenza were also associated with severe illness from influenza A(H1N1) 2009 infection [6,12-20]. However, these studies differ to our study in both the study design and outcomes, making valid comparisons practically impossible. Most of these studies were case-based studies without a control group. Besides, they mainly focussed on risk factors for severe outcomes of influenza, while we studied risk factors for symptomatic infection. Frequent hand washing appeared to have a protective effect on symptomatic infection. Assuming that daily hand washing frequency is a proxy of the hand hygiene in general, this finding suggests that hand hygiene may reduce the transmission of pandemic influenza virus. This is in agreement with recent studies suggesting that hand hygiene prevents transmission of influenza A(H1N1) 2009 virus in households and crowded communities [21-24]. However, heightened attention to hygienic behaviour for controls facing a diseased relative or friend may have caused recall bias in the present study. The results of these analyses could have been influenced by differences in the severity of disease between extramural and hospitalised patients, but exclusion of hospitalised patients led to similar results.

Based on serological responses of the controls included in our study, the secondary transmission rate was over 20%. This is in line with Cowling et al. [23], reporting a fourfold or greater rise in antibody titer in about one fifth of the household contacts, although no distinction was made between pandemic and seasonal influenza virus in that study. The persistently high titers found in four controls in our study, might indicate that they had been exposed to the same source as their case. For at least one control this assumption is supported by a positive PCR at inclusion in the study, although a cross reactive response can not be excluded.

The addition of the FF100 cases and contact approach to the comprehensive assessment of the Dutch pandemic preparedness plan was based on the experiences of the 2003 outbreak of influenza A(H7N7) among poultry in the Netherlands [7,8]. Rapid and structured collection of detailed epidemiological, clinical, virological and immunological data appeared to be essential for the management of control activities and for communication. The inclusion of both cases with relatively mild symptomatic infection and of close contacts is a strength of the FF100 cases and contacts approach. Most studies on influenza A(H1N1) 2009 virus so far concern case-series, mainly including severe cases and most studies on risk factors focused on a serious outcome of disease caused by influenza A(H1N1) 2009 infection [6,12-16,25,26]. Moreover, the inclusion of contacts improved the insight in both risk factors for symptomatic infection in the general Dutch population and in the transmission of pandemic influenza A(H1N1) virus to close contacts.

Nevertheless, the course of the 2009 pandemic and the resulting workload made it impossible to include the intended first few hundred cases and contacts in a limited period of time, which is a substantial limitation of our study. Since the influenza A(H1N1) 2009 virus had already spread internationally before it was recognised, the implementation of containment and mitigation measures was practically impossible, resulting in a rapid increase of the number of cases worldwide [27]. Following the restriction of the mandatory notification in the Netherlands on the 15^th^ of August 2009 [6], only a relatively small number of cases and controls were available for inclusion in the study. Moreover, the public perception of the pandemic changed when it became clear that the majority of patients developed mild disease, and therefore patients were less willing to participate in the study. This is in line with findings in the FF100 cases project in the UK. McLean et al. [28] also showed that initially almost all laboratory-confirmed cases were included in the ‘first few 100 project’ in the UK, but as the case numbers began to increase, the proportion of cases included also decreased.

Nevertheless, the UK FF100 project did succeed to identify key clinical and epidemiological characteristics of infection with the pandemic influenza A(H1N1) virus in near real-time, although the lack of controls suggests that some caution is needed in the interpretation. The higher number of participants might partly be explained by the significant pandemic spring wave in England compared to the Netherlands, which provided many more opportunities for PHS’s to approach people. Also, the absence of the inclusion of controls would make approaching and consenting a more smooth process.

We acknowledge there are some further limitations to our design. First, questionnaire data were used to report patient characteristics and to measure exposure to potential risk factors. This could be less reliable compared with observational data. Moreover, heightened attention to the cause of their complaints by cases may have caused recall bias. Secondly, we cannot rule out the possibility that controls were in the incubation period for infection, even though they had no respiratory complaints at the moment of inclusion. This, in combination with the limited sample size, might have diluted the investigated relations between symptomatic disease and patient characteristics as well as potential risk factors.

Despite the slow and limited recruitment, the FF100 cases and contact approach is well-suited for rapid and detailed collection of epidemiological, clinical, virological and immunological data, and is a necessary addition to case based data. This is also shown by the FF100 cases project in the UK [28]. The UK was one of the first European countries affected and experienced a substantial first wave in spring and summer 2009. Their FF100 cases project was rapidly established and captured information on almost 400 of the first UK cases in the first 7 weeks of the pandemic; 37% of all cases reported in that period [28].

For future pandemics we therefore suggest that several countries share the same comprehensive baseline study protocol to rapidly collect detailed epidemiological, clinical, virological and immunological data of the first few hundred cases as well as their close contacts. This will facilitate the possibility pooling the data, and therefore increase the number of both cases and close contacts resulting in more timely data and more power to strengthen novel findings. Similar European projects are already initiated, like EURO-MOMO monitoring the excess mortality and ECDC I-MOVE monitoring the influenza vaccine effectiveness [29-32]. This will require revision and harmonisation of the pandemic preparedness plans, which has to be elaborated in the inter-pandemic period.

## Conclusion

The labour-intensive design of the FF100 cases and contact approach resulted in a limited recruitment. However the findings of our study were supplementary to those of case based studies and important to guide control activities, to underpin policy decisions, and for communication. Our study showed an increased risk for symptomatic infection in cases with underling non-respiratory disease, a protective effect of frequent hand washing and a secondary transmission rate of about 20%. To increase timeliness and power during future pandemics we suggest pooling the key epidemiological, clinical, virological and immunological data for both the first few hundred cases and their close contacts of several countries, to make it possible to collect the required data in a relatively short time period. Collaborative pooling of the data from several countries may significantly increase the scientific and public health utility of the FF100 approach.

This study was financially supported by the Ministry of Health, Welfare and Sport (the Netherlands) and by The Netherlands Organisation for Health Research and Development Funding (ZonMw).

## Competing interests

The authors declare that they have no competing interest.

## Authors’ contribution

AvG participated in the design and coordination of the study, performed the statistical analyses and had the lead in drafting the manuscript. MvdS participated in the design and coordination of the study, in performing the statistical analyses and drafting the manuscript. AM participated in the design and coordination of the study, drafting the manuscript and was responsible for the virological and serological assays. IF participated in the design and coordination of the study, in performing the statistical analyses and drafting the manuscript. GD participated in the coordination of the study as project leader of the network of general practices. JR participated in the coordination of the study, and was responsible for the serological assays. MR participated in the design of the study. JP participated in the design and coordination of the study with respect to the academic centers. LI participated in the design and coordination of the study. FS participated in drafting the manuscript and headed the network of general practices. MvdL participated in the design and coordination of the study, and in drafting the manuscript, and was responsible for the virological assays. All authors read and approved the final manuscript.

## Dutch ZonMW influenza A(H1N1) 2009 consortium: list of members

L van Asten (RIVM, Bilthoven, the Netherlands), D Baas (RIVM, Bilthoven, the Netherlands), D Beaujean (RIVM, Bilthoven, the Netherlands), J van Beek (RIVM, Bilthoven, the Netherlands), R van Binnendijk (RIVM, Bilthoven, the Netherlands), C Boucher (Erasmus Medical Center, Rotterdam, the Netherlands), M van Boven (RIVM, Bilthoven, the Netherlands), JC Braspenning (Radboud University Nijmegen Medical Centre, Nijmegen, the Netherlands), M Bults (Radboud University Nijmegen Medical Centre, Nijmegen, the Netherlands), R Coutinho (RIVM, Bilthoven, the Netherlands), F Dijkstra (RIVM, Bilthoven, the Netherlands), J van Dissel (Leiden University Medical Center, Leiden, the Netherlands), T Donker (RIVM, Bilthoven, the Netherlands), GA Donker (NIVEL, the Netherlands), R Fouchier (Erasmus Medical Center, Rotterdam, the Netherlands), P de Fraaij (Erasmus Medical Center, Rotterdam, the Netherlands), IHM Friesema (RVIM, Bilthoven, the Netherlands), AB van Gageldonk-Lafeber (RIVM, Bilthoven, the Netherlands), R Grol (Radboud University Nijmegen Medical Centre, Nijmegen, the Netherlands), F Heijningen (RIVM, Bilthoven, the Netherlands), W van der Hoek (RIVM, Bilthoven, the Netherlands), A van den Hoek (Public Health Service of Amsterdam and LOI, Amsterdam, the Netherlands), M Hooiveld (NIVEL, Utrecht, the Netherlands), M Hulscher (Radboud University Nijmegen Medical Centre, Nijmegen, the Netherlands), J IJzermans (NIVEL, Utrecht, the Netherlands), L Isken (RIVM, Bilthoven, the Netherlands), M de Jong (Academical Medical Centre – University Amsterdam, the Netherlands), A. Kroneman (RIVM, Bilthoven, the Netherlands), Can Kesmir (Utrecht University Medical Center, Utrecht, the Netherlands), F van der Klis (RIVM, Bilthoven, the Netherlands), T van 't Klooster (RIVM, Bilthoven, the Netherlands, M Koopmans (RIVM, Bilthoven, the Netherlands), M Kretzschmar (RIVM, Bilthoven, the Netherlands), IM van der Lubben (RIVM, Bilthoven, the Netherlands), M Mak (RIVM, Bilthoven, the Netherlands), J van der Meer (Radboud University Nijmegen Medical Centre, Nijmegen, the Netherlands), A Meijer (RIVM, Bilthoven, the Netherlands), JJ van Oosterheert (Utrecht University Medical Center, Utrecht, the Netherlands), A Osterhaus (Erasmus Medical Center, Rotterdam, the Netherlands), J Prins (Academical Medical Centre – University Amsterdam, the Netherlands), J Reimerink (RIVM, Bilthoven, the Netherlands), R Riesmeijer (RIVM, Bilthoven, the Netherlands), G Rimmelzwaan (Erasmus Medical Center, Rotterdam, the Netherlands), MAB van der Sande (RIVM, Bilthoven, the Netherlands), F Schellevis (NIVEL, Utrecht, the Netherlands), M Schutten (Erasmus Medical Center, Rotterdam, the Netherlands), J van Steenbergen (RIVM, Bilthoven, the Netherlands), A Steens (RIVM, Bilthoven, the Netherlands), MAJB Tacken (Radboud University Nijmegen Medical Centre, Nijmegen, the Netherlands), P Teunis (RIVM, Bilthoven, the Netherlands), A Timen (RIVM, Bilthoven, the Netherlands), M van der Velden (iResearch, Berg en Dal, the Netherlands), R Verheij (NIVEL, Utrecht, the Netherlands), L Vinck (RIVM, Bilthoven, the Netherlands), J Wallinga (RIVM, Bilthoven, the Netherlands), A Westerhof (RIVM, Bilthoven, the Netherlands), L Wielders (RIVM, Bilthoven, the Netherlands), CC van den Wijngaard (RIVM, Bilthoven, the Netherlands), O de Zwart (Rotterdam-Rijnmond Public Health Service, Rotterdam, the Netherlands).

## References

[B1] Fauci AS Pandemic influenza threat and preparedness Emerg Infect Dis 2006 12 1 73 77 16494721 10.3201/eid1201.050983 PMC3291399

[B2] Guan Y Poon LL Cheung CY Ellis TM Lim W Lipatov AS Chan KH Sturm-Ramirez KM Cheung CL Leung YH H5N1 influenza: a protean pandemic threat Proc Natl Acad Sci U S A 2004 101 21 8156 8161 10.1073/pnas.0402443101 15148370 PMC419573

[B3] Webster RG Govorkova EA H5N1 influenza–continuing evolution and spread N Engl J Med 2006 355 21 2174 2177 10.1056/NEJMp068205 17124014

[B4] Whitley RJ Monto AS Seasonal and pandemic influenza preparedness: a global threat J Infect Dis 2006 194 Suppl 2 S65 S69 17163390 10.1086/507562

[B5] Hahne S Donker T Meijer A Timen A Van Steenbergen J Osterhaus A van der Sande M Koopmans M Wallinga J Coutinho R Epidemiology and control of influenza A(H1N1)v in the Netherlands: the first 115 cases Euro Surveill 2009 14 27 19267 19589332 10.2807/ese.14.27.19267-en

[B6] Klooster TM Wielders CC Donker T Isken L Meijer A van den Wijngaard CC van der Sande MA van der Hoek W Surveillance of hospitalisations for 2009 pandemic influenza a(h1n1) in the netherlands, 5 june - 31 december 2009 Euro Surveill 2010 15 2) 19461 20085691 10.2807/ese.15.02.19461-en

[B7] Fouchier RA Schneeberger PM Rozendaal FW Broekman JM Kemink SA Munster V Kuiken T Rimmelzwaan GF Schutten M Van Doornum GJ Avian influenza a virus (h7n7) associated with human conjunctivitis and a fatal case of acute respiratory distress syndrome Proc Natl Acad Sci U S A 2004 101 5 1356 1361 10.1073/pnas.0308352100 14745020 PMC337057

[B8] Koopmans M Wilbrink B Conyn M Natrop G Nat H Vennema H Meijer A van Steenbergen J Fouchier R Osterhaus A Transmission of h7n7 avian influenza a virus to human beings during a large outbreak in commercial poultry farms in the netherlands Lancet 2004 363 9409 587 593 10.1016/S0140-6736(04)15589-X 14987882

[B9] Friesema IH Meijer A Van Gageldonk-Lafeber AB van der Lubben IM van Beek J Donker GA Prins JM de Jong MD Boskamp S Isken LD Koopmans MP van der Sande MAB Dutch ZonMW Influenza A(H1N1) 2009 consortium Course of pandemic influenza A(H1N1)2009 virus infection in Dutch patients Influenza and other respiratory viruses 2012 6 3 e16 20 10.1111/j.1750-2659.2012.00347.x 22372759 PMC4941673

[B10] Meijer A Beerens A Claas E Hermans M de Jong A Molenkamp R Niesters H Overduin P Rossen J Schuurman R Preparing the outbreak assistance laboratory network in the netherlands for the detection of the influenza virus a(h1n1) variant J Clin Virol 2009 45 3 179 184 10.1016/j.jcv.2009.06.003 19540155

[B11] Steens A Waaijenborg S Teunis PF Reimerink JH Meijer A van der Lubben M Koopmans M van der Sande MA Wallinga J van Boven M Age-dependent patterns of infection and severity explaining the low impact of 2009 influenza A (H1N1): evidence from serial serologic surveys in the Netherlands Am J Epidemiol 2011 174 11 1307 1315 10.1093/aje/kwr245 22025354

[B12] Jain S Kamimoto L Bramley AM Schmitz AM Benoit SR Louie J Sugerman DE Druckenmiller JK Ritger KA Chugh R Hospitalized patients with 2009 h1n1 influenza in the united states, april-june 2009 N Engl J Med 2009 361 20 1935 1944 10.1056/NEJMoa0906695 19815859

[B13] Louie JK Acosta M Jamieson DJ Honein MA Severe 2009 h1n1 influenza in pregnant and postpartum women in california N Engl J Med 2010 362 1 27 35 10.1056/NEJMoa0910444 20032319

[B14] Nguyen-Van-Tam JS Openshaw PJ Hashim A Gadd EM Lim WS Semple MG Read RC Taylor BL Brett SJ McMenamin J Risk factors for hospitalisation and poor outcome with pandemic a/h1n1 influenza: united kingdom first wave (may-september 2009) Thorax 2010 65 7 645 651 10.1136/thx.2010.135210 20627925 PMC2921287

[B15] Santa-Olalla Peralta P Cortes-Garcia M Vicente-Herrero M Castrillo-Villamandos C Arias-Bohigas P Pachon-del Amo I Sierra-Moros MJ Risk factors for disease severity among hospitalised patients with 2009 pandemic influenza a (h1n1) in spain, april - december 2009 Euro Surveill 2010 15 38 19667 20929651 10.2807/ese.15.38.19667-en

[B16] Viasus D Pano-Pardo JR Pachon J Campins A Lopez-Medrano F Villoslada A Farinas MC Moreno A Rodriguez-Bano J Oteo JA Factors associated with severe disease in hospitalized adults with pandemic (h1n1) 2009 in spain Clin Microbiol Infec 2011 17 5 738 46 10.1111/j.1469-0691.2010.03362.x 20825436

[B17] Fang L Lin J Chai C Yu Z Risk factors for severe cases of 2009 influenza A (H1N1): a case control study in zhejiang province China. PLoS One 2012 7 3 e34365 10.1371/journal.pone.0034365 PMC3314610 22470561

[B18] Launes C Garcia-Garcia JJ Martinez-Planas A Moraga F Astigarraga I Aristegui J Korta J Salado C Quintana JM Soldevila N 2009 H1N1: risk factors for hospitalization in a matched case–control study Eur J Pediatr 2012 171 7 1127 31 10.1007/s00431-012-1716-6 22430351

[B19] Ward KA Spokes PJ McAnulty JM Case–control study of risk factors for hospitalization caused by pandemic (h1n1) 2009 Emerg Infect Dis 2011 17 8 1409 1416 21801617 10.3201/eid1708.100842 PMC3381572

[B20] Gilca R De Serres G Boulianne N Ouhoummane N Papenburg J Douville-Fradet M Fortin E Dionne M Boivin G Skowronski DM Risk factors for hospitalization and severe outcomes of 2009 pandemic h1n1 influenza in quebec, canada Influenza Other Respi Viruses 2011 5 4 247 255 10.1111/j.1750-2659.2011.00204.x 21651735 PMC4634547

[B21] Aiello AE Murray GF Perez V Coulborn RM Davis BM Uddin M Shay DK Waterman SH Monto AS Mask use, hand hygiene, and seasonal influenza-like illness among young adults: a randomized intervention trial J Infect Dis 2010 201 4 491 498 10.1086/650396 20088690

[B22] Cowling BJ Chan KH Fang VJ Cheng CK Fung RO Wai W Sin J Seto WH Yung R Chu DW Facemasks and hand hygiene to prevent influenza transmission in households: a cluster randomized trial Ann Intern Med 2009 151 7 437 446 19652172 10.7326/0003-4819-151-7-200910060-00142

[B23] Cowling BJ Chan KH Fang VJ Lau LL So HC Fung RO Ma ES Kwong AS Chan CW Tsui WW Comparative epidemiology of pandemic and seasonal influenza a in households N Engl J Med 2010 362 23 2175 2184 10.1056/NEJMoa0911530 20558368 PMC4070281

[B24] Gould D Hand hygiene and facemask use within 36 hours of index patient symptom onset reduces flu transmission to household contacts Evid Based Nurs 2010 13 2 44 10.1136/ebn.13.2.44 20436144

[B25] Fuhrman C Bonmarin I Bitar D Cardoso T Duport N Herida M Isnard H Guidet B Mimoz O Richard JC Adult intensive-care patients with, pandemic influenza a(h1n1) infection Epidemiol Infect 2009 2010 1 8 10.1017/S0950268810002414 20974021

[B26] Siston AM Rasmussen SA Honein MA Fry AM Seib K Callaghan WM Louie J Doyle TJ Crockett M Lynfield R Pandemic 2009 influenza a(h1n1) virus illness among pregnant women in the united states JAMA 2010 303 15 1517 1525 10.1001/jama.2010.479 20407061 PMC5823273

[B27] Smith GJ Vijaykrishna D Bahl J Lycett SJ Worobey M Pybus OG Ma SK Cheung CL Raghwani J Bhatt S Origins and evolutionary genomics of the 2009 swine-origin h1n1 influenza a epidemic Nature 2009 459 7250 1122 1125 10.1038/nature08182 19516283

[B28] McLean E Pebody RG Campbell C Chamberland M Hawkins C Nguyen-Van-Tam JS Oliver I Smith GE Ihekweazu C Bracebridge S Pandemic (h1n1) 2009 influenza in the uk: clinical and epidemiological findings from the first few hundred (ff100) cases Epidemiol Infect 2010 138 11 1531 1541 10.1017/S0950268810001366 20594381

[B29] European monitoring of excess mortality for public health actionhttp://www.euromomo.eu/17370927

[B30] Kissling E Valenciano M Falcao J Larrauri A Widgren K Pitigoi D Oroszi B Nunes B Savulescu C Mazick A "I-move" towards monitoring seasonal and pandemic influenza vaccine effectiveness: lessons learnt from a pilot multi-centric case–control study in europe Euro Surveill 2008 14 44) 19388 19941774

[B31] Mazick A Gergonne B Wuillaume F Danis K Vantarakis A Uphoff H Spiteri G Klooster T Junker C Holmberg M Molbak K Higher all-cause mortality in children during autumn 2009 compared with the three previous years: pooled results from eight European countries Euro Surveill 2010 15 5 19480 20144446

[B32] Valenciano M Kissling E Cohen JM Oroszi B Barret AS Rizzo C Nunes B Pitigoi D Larrauri Camara A Mosnier A Estimates of pandemic influenza vaccine effectiveness in europe, 2009–2010: results of influenza monitoring vaccine effectiveness in europe (i-move) multicentre case–control study PLoS Med 2011 8 1 e1000388 10.1371/journal.pmed.1000388 21379316 PMC3019108

